# The Natural History of Hearing Disorders in Asymptomatic Congenital Cytomegalovirus Infection

**DOI:** 10.3389/fped.2020.00217

**Published:** 2020-05-05

**Authors:** Serena Salomè, Antonietta Giannattasio, Rita Malesci, Elio Marciano, Pasquale Dolce, Giuseppe Portella, Grazia Isabella Continisio, Pasquale Di Costanzo, Eleonora Capone, Clara Coppola, Letizia Capasso, Francesco Raimondi

**Affiliations:** ^1^Division of Neonatology, Department of Translational Medical Sciences, University of Naples “Federico II”, Naples, Italy; ^2^Pediatric Emergency Department, AORN Santobono-Pausilipon, Naples, Italy; ^3^Unit of Audiology, Department of Neurosciences, Reproductive and Odontostomatologic Sciences, University of Naples “Federico II”, Naples, Italy; ^4^Department of Public Health, University of Naples “Federico II”, Naples, Italy; ^5^Division of Clinical Pathology, Department of Translational Medical Sciences, University of Naples “Federico II”, Naples, Italy

**Keywords:** congenital CMV infection, sensorineural hearing loss, asymptomatic infection, fluctuating SNHL, delayed SNHL

## Abstract

**Background and Aim:** Cytomegalovirus (CMV) is the main cause of congenital infection in developed countries leading to deafness but the burden of sensorineural hearing loss (SNHL) in asymptomatic children remains incompletely characterized. Aim of this study was to evaluate the long-term audiological outcome in this group of patients.

**Methods:** Consecutive neonates with congenital CMV infection were followed from 2002 to 2018. Patients were considered asymptomatic if free from any clinical and instrumental impairment at referral and underwent serial clinical exams, audiological evaluations and CMV-PCR determinations.

**Results:** A cohort of 258 children was analyzed and the disease onset was asymptomatic in 125 (48%) infants. Among these, we studied 102 patients with a follow-up longer than 1 year and a median observation period of 2.8 years (range: 1–10.3 years). No patient developed a stable delayed SNHL but only 14 (14%) presented a variable hearing impairment, seven of which bilateral. The unstable SNHL was mild in 12 infants and moderate in two. Patients with fluctuating SNHL had significantly higher urine viral load (*p* 0.002) and more often positive viremia (*p* 0.015) than babies with stable normal hearing.

**Conclusions:** CMV infected, asymptomatic neonates have a low risk of transient SNHL later in infancy. Positive viremia and high urine viral load at onset are significant risk factors for delayed fluctuating SNHL. These data are relevant for an appropriate follow up plan of these patients.

## Introduction

Cytomegalovirus (CMV) is the most common cause of congenital infection (1) and one of the most frequent non-genetic cause of sensorineural hearing loss (SNHL) (2–6). The hearing impairment may be fluctuating in time or progressive up to require cochlear implants (7, 8). Congenital CMV (cCMV) infection is defined by the presence of viral DNA in the urine though up to 90% of neonates with viruria show no apparent abnormalities and normal hearing (9). The definition of asymptomatic cCMV infection, however, is not univocal and some researchers have included in this group infants with SNHL (8, 10). In addition, the heterogeneity of diagnostic methods and hearing evaluation may contribute to explain the variable prevalence of SNHL reported in babies with symptomatic cCMV in literature (11, 12). The SNHL ranges from 7 to 27% overall while late/delayed hearing loss would occur in 11.1 to 18.2% with a median age at onset of 44 months (from 24 to 182) (10, 13). Recent consensus recommendations for prevention, diagnosis and therapy of cCMV infection have defined as purely asymptomatic the infant with no apparent abnormalities to suggest cCMV disease and normal hearing (9). The aim of the present study is to evaluate the long term audiological outcome from a relatively large population of cCMV infected infants who have been previously assessed as asymptomatic, according to this more recent and strict definition. A reliable investigation of these patients is important for an appropriate follow up program.

## Materials and Methods

### Study Population

This is a prospective study conducted at the Perinatal Infection Unit of the University Federico II of Naples, a tertiary care hospital with a dedicated multidisciplinary team. Inborn neonates born from 2002 to 2018 or referred to the Unit for maternal CMV seroconversion were considered eligible. Congenital infection was defined as viral DNA detection in urine by polymerase chain reaction assay within the first 3 weeks of life. Viral DNA detection in the blood and in urine was performed using an automated QIAsimphony SP platform performing all steps of the purification procedure and preparation for CMV DNA amplification, in combination with the Artus CMV QS-RGQ Kit on Rotor-Gene® Q system, accordingly to the manufacturer's instructions. Infants with no proof of CMV infection or with other congenital infections were excluded from the study. Clinical and instrumental evaluations are described in Table 1.

**Table 1:** Clinical and instrumental evaluations.

Initial evaluation (within the first 4 weeks from birth)	• Physical examination (including weight, length and head circumference measurements) • Complete blood count • Liver and kidney function tests • CMV viral load in plasma and urine and cerebrospinal fluid • One or more neuroimaging studies • Ophthalmologic evaluation • Hearing evaluation
3 months	• Clinical and laboratory evaluations (including CMV viral load in plasma and urine) • Fundoscopy • Hearing evaluation • Neurological examination • Psychomotor development test
Every 6 months up to 2 years	• Clinical and laboratory evaluations (including CMV viral load in plasma and urine) • Fundoscopy • Hearing evaluation • Psychomotor development test
Every year up to 6 years	• Clinical and laboratory evaluations (including CMV viral load in plasma and urine) • Fundoscopy • Hearing evaluation • Psychomotor development test

### Neuroimaging Studies

Cranial ultrasonography (US) was performed by an experienced neonatologist, who was blinded to the clinical data, using the Philips HD11 ultrasound imaging platform with an 8.5–12.4 MHz transducers (Microconvex and Phased Array transducers). Abnormal US was defined as presence of intracranial calcifications, ventriculomegaly, pseudocysts, or cerebellar lesions. Isolated candlestick lentriculostriated vasculopathy without other abnormalities was not considered as a sign of symptomatic cCMV infection (14). Head CT was performed with lower mA and kVp settings in order to reduce the radiation exposure (Philips). CT scans were interpreted by an experienced pediatric neuroradiologist and findings were classified as normal or abnormal according to the presence of intracranial calcifications, ventriculomegaly, or suspected neuronal migration disorders (15). MRI was performed with a superconductive 1.5 T system (Philips Healthcare, The Netherlands). Sedation was performed with midazolam while the induction to the anesthesia was obtained with sevoflurane and oxygen, in order to minimize motion artifacts. A neuroradiologist with a specific expertise in pediatric diseases interpreted brain MRIs. Intravenous contrast medium was not used in any of the patients.

### Audiological Assessment

Enrolled patients underwent serial standard audiological evaluations to detect SNHL within the 1st month of life, every 3–6 months until the age of 3 years and then every 6–12 months later on. Hearing function was evaluated by TEOAE (transitory evoked otoacoustic emissions) at birth and by automatic ABR (auditory brainstem response) within the 1st month of life. In case of negative results, a click ABR and, according to the age of patient, pure tone audiometry and other specific tests, such as DPOAE (distortion product otoacoustic emissions), tympanometry, etc. were performed. Before 2007 the early audiological assessment was carried out in the 1st month of life through click ABR and tympanometry. Patients were examined more frequently in case of hearing loss, as established by the audiologist. In order to describe SNHL, the poorer hearing ear criterion was adopted as the most accurate to describe cCMV related damage.

SNHL was defined as air conduction thresholds >20 decibels (dB), in conjunction with normal bone conduction threshold and normal middle-ear function. Hearing loss was classified using the BIAP criteria (16), as reported in Table 2. We classified SNHL as *monolateral* if it was present in one ear, or *bilateral* if it was present in both ears. A *delayed-onset* hearing loss was defined when detected after ≥1 assessments with normal hearing. *Progressive* hearing loss was defined as a worsening of the auditory threshold with 10 dB or more in successive assessments. *Stable* hearing loss was defined when there was no change between the first detection of SNHL and the last assessment. *Improvement* of hearing loss was defined when hearing thresholds improved with 10 dB or more. A *fluctuating* hearing loss was defined as an improvement of at least 10 dB after a previous worsening or a worsening after a previous improvement between consecutive assessments. Since middle ear disorders can cause transient conductive hearing loss, we excluded assessments with tympanometry type B routinely performed.

**Table 2:** SNHL definition.

Mild	21–40 dB
Moderate 1st degreeModerate 2nd degree	41–55 dB56–70 dB
Severe 1st degreeSevere 2nd degree	71–80 dB81–90 dB
Very severe 1st degreeVery severe 2nd degreeVery severe 3rd degree	91–100 dB101–110 dB111–119 dB
Total HL	>120 dB

Patients were classified as symptomatic at onset if presenting with one or more of the following: lethargy, seizures, poor suck, microcephaly (head circumference <2 SD below the mean for age and birth weight), chorioretinitis, hepatosplenomegaly, petechiae, elevated serum transaminase levels, cholestasis, thrombocytopenia (<100,000 platelets/mm^3^), hearing impairment, and abnormal findings on central nervous system (CNS) imaging evaluation. US and MRI scan, and CT in past years (17), evaluated CNS abnormalities (calcifications, neuronal migration disorders, cerebral, and cerebellar volume loss, ventriculomegaly, white matter disease).

Patients were defined as asymptomatic if free from all signs listed above soon after birth. Data were recorded on a standardized database. The Ethics Committee of our Institution (Comitato Etico “Carlo Romano,” Università Federico II di Napoli) approved this study (protocol number 274/16).

### Data Analysis

All data analyses were performed using the statistical platform R (R Core Team 2018). Numerical variables were described using mean ± standard deviation (SD) or median with range [min; max] while categorical variables were summarized using absolute frequencies and percentages. Between-groups differences were, accordingly, assessed by the unpaired *T*-test or the Mann-Whitney *U*-tests for numerical variables and the Chi square test, or the Fisher exact test if appropriate, for categorical factors. Times to event variables were analyzed using the Kaplan Meier methods and compared between groups using the log-rank test. Univariate Cox regression models were used to estimate Hazard Ratio (HR) with the corresponding 95% Confidence Intervals (95% C.I.). All test were 2-tailed and *p* < 0.05 were considered statistically significant.

## Results

### Study Population

**Figure 1:**
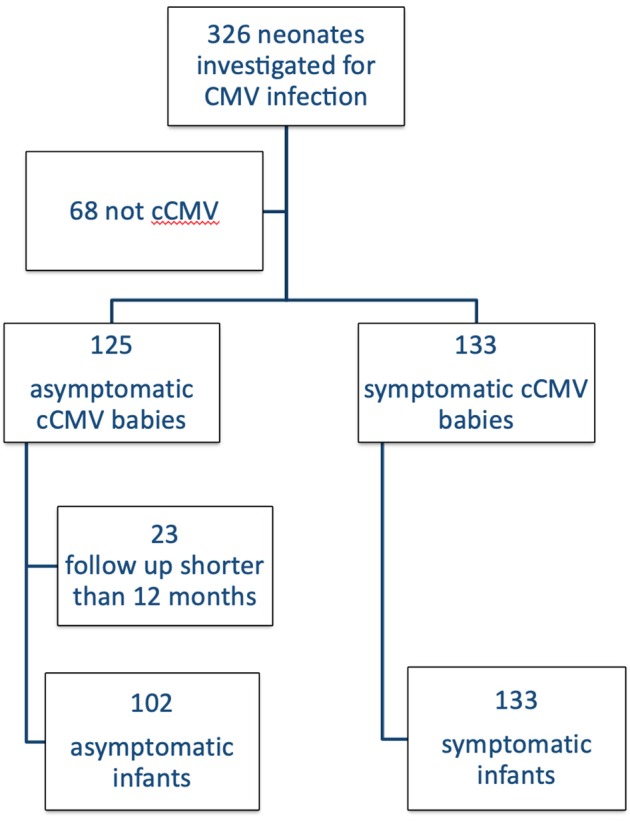
Study population.

Out of a total of 326 patients referred to our Unit, 258 received a diagnosis of cCMV infection and 125 (48%) were classified as asymptomatic. One hundred two babies had a follow up longer than 1 year and met the inclusion criteria (Figure 1). Their general characteristics are summarized in Table 3. Table 4 summarizes our main results.

**Table 3:** Demographic and clinical features at onset in patients with cCMV asymptomatic infection.

Features^*^	**Asymptomatic patients (*n* = 102)**
Males	55 (54%)
Age at last observation (years)	3.3 ± 1.8 (1–10.3)
Gestational age (weeks)	38 (31–42)
Number of preterm infants (<37 wks gestational age)	10 (10%)
Birth weight (grams)	3,021 ± 560 (1,310–4,400)
Head circumference at birth (cm)	33.7 ± 1.5 (28–37)
Apgar score at 5 min	9 (7–10)

* *Values are expressed as absolute frequencies and percentages, mean ± standard deviation and range or median with range, as appropriated*.

**Table 4:** SNHL and laboratory features at onset in patients with cCMV infection.

Features^*^	**Asymptomatic patients (*n* = 102)**	**Symptomatic patients (*n* = 133)**	***p***
SNHL	0 (0%)	85 (64%) Monolateral 29 (22%) Bilateral 56 (42%)	–
Plasma CMV DNA (positive)	49 (51%)	86 (62%)	*p* = 0.105
Plasma CMV DNA (copies/ml)	2,330 [95% C.I. 750–4,092] (range 178–49,600)	2,100 [95% C.I. 1,100–10,725] (range 160–11,300,000)	*p* = 0.16
Urinary CMV DNA (copies/ml)	776,349 [95% C.I. 69,500–3,135,000] (range 1,000–111,000,000)	243,101.5 [95% C.I. 100,000–1,982,500] (range 160–572,000,000)	*p* = 0.333
CSF CMV DNA (positive)	0/42 (0%)	13/84 (15%)	*p = 0.005*
CSF CMV DNA (copies/ml)	0	1,423 [95% C.I. 925–14,183] (range 160–58,000)	–

* *Values are expressed as absolute frequencies and percentages, mean ± standard deviation and range or median with range, as appropriate*.

The overall incidence of SNHL at onset in symptomatic patients was 64%: 29 monolateral cases (22%) and 56 bilateral ones (42%). According to our definition of asymptomatic cCMV infection, we had no hearing loss at onset in this group.

Comparing symptomatic and asymptomatic patients according to the viral presence at onset, we found no statistical difference in viremia (considering it as positive or negative [*p* = 0.105]) nor considering the CMV blood load (*p* = 0.16) or in viruria (*p* = 0.333). A lumbar puncture was part of the standard initial evaluation of the asymptomatic neonate until December 2010. The evaluation of cerebrospinal fluid (CSF) was available for 42 asymptomatic and 84 symptomatic. No virus was detected in the former group while 13 symptomatic infants (15%) had a positive CMV in the CSF (*p* = 0.005). In the asymptomatic group, time to blood viral clearance was 104 days (95% C.I. 83–163 days, range: 11–659 days) in blood and 1,437 days (95% C.I. 1,233–2,756 days, range: 230–2,756 days) in urine. At the last observation 51 patients (51%) had no virus detectable in urine. Viral clearance in symptomatic babies depends on the antiviral therapy and is not reported here.

**Figure 2:**
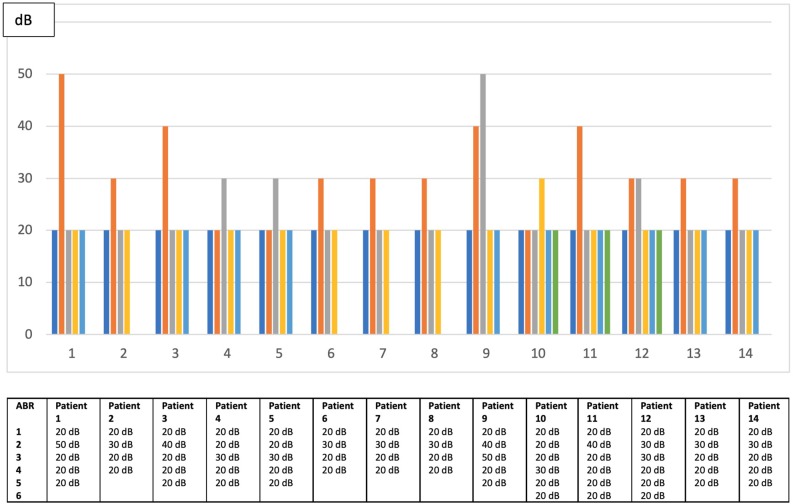
Fluctuations of hearing threshold among patients with delayed unstable hearing loss in worst ear.

The median number of audiological assessments to be taken in account was 4 (range: 3–9). In fact, we excluded assessments showing middle ear disorders causing transient conductive hearing loss. No asymptomatic infant developed a stable delayed hearing loss and 88 patients (86%) presented a normal hearing function at all audiological assessments. Only 14 patients (14%) presented a fluctuating hearing impairment that was bilateral in seven babies. Their transient dysfunction was classified as mild in 12 children and moderate first degree in the other two (the minimal and maximal thresholds were, respectively 20 and 50 dB) as shown in Figure 2. None of the patients with fluctuating SNHL had a sufficient hearing loss to justify hearing aids or cochlear implant. When comparing babies with fluctuating SNHL to those with a stable normal hearing throughout follow up, the former group had a significant higher number of infants with positive viremia and with a greater urinary viral load (Table 5). None of the asymptomatic infants developed later sequelae such as neurologic disorders, visual loss, thrombocytopenia, or liver disease during the follow-up period.

**Table 5:** SNHL in asymptomatic patients.

Features^*^	**Stable normal hearing (88)**	**Fluctuating SNHL (*n* = 14)**	***p***
Plasma CMV DNA at onset (positive)	38 (45%)	11 (84.6)	*p* = 0.015
Plasma CMV DNA at onset (copies/ml)	2,630 [1,000–4,120.5] (178–28,800)	1,870 [555–3,800] (263–49,600)	*p* = 0.544
Urinary CMV DNA at onset (copies/ml)	472,500 [47,250–2,582,192.5] (1,000–111,000,000)	3,250,000 [1,890,000–14,950,000] (45,000–36,300,000)	*p* = 0.002
Time to blood viral clearance (months)	2.9 (2.2–6.21)	3.8 [3.1–na]	*p* = 0.547
Time to urine viral clearance (months)	3.8 [3.4–7.6]	4.9 [2.8–n.a.]	*p* = 0.829

* *Values are expressed as absolute frequencies and percentages, mean ± standard deviation and range or median with range, as appropriate*.

## Discussion

In our cohort of 102 cCMV infected, purely asymptomatic infants, no one developed a stable SNHL after a mean follow up period of 3.3 years, with a median of 2.8 years. The quality of the result is strengthened by the investigation of babies free from hearing impairment shortly after birth, with an homogeneous clinical and instrumental follow-up period that included scrupulous search for middle-ear problems so that hearing fluctuations could not be due to transient, CMV-unrelated causes (9). Our data on SNHL in asymptomatic patients are of relevance in view of cCMV screening programs being implemented worldwide. It is likely that more asymptomatic infants will be identified and there is a need for an evidence-based classification and management of these infants.

Our results, however, do not confirm previous observations were delayed SNHL seemed to be a relatively frequent event. Among the possible explanations, the precise definition of an asymptomatic, cCMV infected neonate is, probably, the most relevant.

In an earlier report by Fowler et al. (3), the definition of asymptomatic case is not clear and 50% of their infants were affected by prematurity, a known independent risk factor for SNHL. Later observations by Foulon et al. considered as asymptomatic infants affected by other postnatal risk factors for SNHL (e.g., bacterial meningitis after the newborn period) (18) or regardless their hearing disabilities (19).

In a recent multicenter study, Lanzieri et al. classified as asymptomatic (8) also patients who would now be classified as isolated hearing impairment at onset; in this case the risk of delayed-onset SNHL was significantly greater among patients with monolateral congenital/early-onset loss than those with bilateral normal hearing at birth. In the latter group, some babies who did develop a delayed SNHL had some neuroimaging abnormalities and would not have been now labeled as truly asymptomatic. As in our study, a normal bilateral hearing function in babies free from any detectable CMV sign or symptom is a significant predictor of a subsequent more favorable hearing outcome.

Recently, Faure-Bardon et al. (20) showed that sequelae were present only in the progeny of mothers who seroconverted in the first trimester of pregnancy.

Unfortunately, we were unable to replicate their observation since details on maternal seroconversion during gestation were available in too few cases to draw reliable conclusions.

In our series, the presence of CMV in the blood and its urinary load did not discriminate between symptomatic and asymptomatic infants. In the latter group, however, these two variables were significantly associated with a non-severe and transient hearing impairment. Whether the viral burden is a reliable SNHL predictor in infants with isolated hearing impairment or with frank cCMV symptoms at onset is currently debated (21–26). We speculate that the presence of SNHL with or without other symptoms may be due to a rather complex interaction of factors other than the simple viral load around birth in blood or urine. It is noteworthy the absence of CMV in all the available CSF samples from the asymptomatic cohort.

We acknowledge that a longer average duration of our follow up would have strengthened our results. However, it has to be underlined that 18% of our cohort has a follow-up longer than 5 years.

In conclusion, cCMV infected infants free from clinical symptoms and SNHL within the 1st month of life carry a very low risk of delayed hearing impairment. Our cohort identifies a stage of the interaction between the virus and the human neonate where a prolonged follow up program seems unnecessary.

## Data Availability Statement

The datasets for this study will not be made publicly available because it is confidential patient data. Any requests to access the datasets should be directed to Serena Salomè, serena.salome@unina.it.

## Ethics Statement

The study was conducted in accordance with Helsinki Declaration as revised in 2013. The protocol was approved by the Ethics Committee of the Azienda Federico II (protocol number 274/16 of 2016). Parents of participants provided their written informed consent to participate in this study.

## Author Contributions

SS designed the study, analyzed data, interpreted results, and drafted the article. AG, PD, LC, and GC contributed to the revision of draft and interpretation of data. CC and EC were involved in the acquisition of data. RM and EM performed and revised audiological outcomes. GP performed the virological analysis. PD analyzed data. FR conceived the study and revised the article critically for important intellectual content. All authors read and approved the final version of the manuscript.

## Conflict of Interest

The authors declare that the research was conducted in the absence of any commercial or financial relationships that could be construed as a potential conflict of interest.
